# Facilitating Responsible Gambling: The Relative Effectiveness of Education-Based Animation and Monetary Limit Setting Pop-up Messages Among Electronic Gaming Machine Players

**DOI:** 10.1007/s10899-012-9340-y

**Published:** 2012-10-23

**Authors:** Michael J. A. Wohl, Sally Gainsbury, Melissa J. Stewart, Travis Sztainert

**Affiliations:** 1 Department of Psychology, Carleton University, 1125 Colonel By Drive, Ottawa, ON K1S 5B6 Canada; 2 Centre for Gambling Education and Research, Southern Cross University, PO Box 157, Lismore, NSW 2480 Australia; 3 Department of Psychology, Dalhousie University, 1355 Oxford Street, Halifax, Nova Scotia B3H 4JI Canada

**Keywords:** Limit setting, Limit adherence, Erroneous cognitions, Pop-up message, Education, Animation, Responsible gambling

## Abstract

Although most gamblers set a monetary limit on their play, many exceed this limit—an antecedent of problematic gambling. Responsible gambling tools may assist players to gamble within their means. Historically, however, the impact of such tools has been assessed in isolation. In the current research, two responsible gambling tools that target adherence to a monetary limit were assessed among 72 electronic gaming machine (EGM) players. Participants watched an educational animation explaining how EGMs work (or a neutral video) and then played an EGM in a virtual reality environment. All participants were asked to set a monetary limit on their play, but only half were reminded when that limit was reached. Results showed that both the animation and pop-up limit reminder helped gamblers stay within their preset monetary limit; however, an interaction qualified these main effects. Among participants who did not experience the pop-up reminder, those who watched the animation stayed within their preset monetary limits more than those who did not watch the animation. For those who were reminded of their limit, however, there was no difference in limit adherence between those who watched the animation and those who did not watch the animation. From a responsible gambling perspective, the current study suggests that there is no additive effect of exposure to both responsible gambling tools. Therefore, for minimal disruption in play, a pop-up message reminding gamblers of their preset monetary limit might be preferred over the lengthier educational animation.

## Introduction

Liberalization of gambling policies and cross-jurisdictional competition has led to significant global increases in gambling in both land-based and Internet forms. Electronic gaming machines (EGMs) are widely available in casinos and local venues in many parts of Canada, Australia, the United States, United Kingdom, Europe and an increasing number of Asian and South American countries. The expansion of gambling is generally justified by arguments of economic benefits, providing a regulated environment, and using taxes and profits to make positive community contributions (Economopoulos 2007; Smith et al. 2011). However, it has been argued that gambling-related harms may outweigh its benefits (Orford et al. 2009; Smith et al. 2011; Toce-Gerstein and Gerstein 2007; Volberg 2001). As a result, we, like others (Blaszczynski et al. 2004; Gainsbury 2011), suggest there is a need to implement empirically tested tools that assist gamblers to engage in play responsibly.

Responsible gambling strategies aim to encourage players to gamble within their means,^1^ whilst having minimal disruption for recreational players (Blaszczynski et al. 2004). A key strategy for promoting responsible gambling is setting a monetary limit on play (i.e., how much money can be lost in a given session; Brown and Newby-Clark 2005; Gollwitzer et al. 2004). This is because many gamblers have a problem limiting the money they spend on gambling. The need to promote monetary limit adherence is especially important as failure to do so increases alongside symptoms of pathological gambling (Nower and Blaszczynski 2010; Schellinck and Schrans 2002; Wohl et al. 2010), which is in turn associated with a wide range of problems including psychological distress and co-morbid disorders, poor health, employment difficulties, family breakdown, crime, and suicide (see Neal et al. 2005). In Australia alone, problematic gambling is estimated to yield social costs of AUD$4.7 billion annually (Productivity Commission 2010).

Recognizing the need to promote limit setting and adherence, Wohl et al. (2010) created a cognitively simply animation that educated gamblers about how EGMs function and the benefits of setting a monetary limit on play. They showed that this animation not only facilitated limit setting, but limit adherence as well. Other research has shown responsible gambling benefits of pop-up messages on EGMs that inform the gamblers, among other things, about the probability of wins (see Monaghan 2008; Monaghan and Blaszczynski 2007; Monaghan and Blaszczynski 2010a) or directly asks the gambler to set and record a monetary limit prior to play (Stewart and Wohl in press). The efficacy of these responsible gambling tools are typically designed and assessed in isolation. In gambling venues, however, gamblers are typically exposed to an array of such responsible gambling messages simultaneously. The possible interactive effect of such exposure is not yet known.

The current research aims to extend the extant literature on responsible gambling tools by assessing the consequence of being exposed to more than one responsible gambling tool within a given gambling session. Specifically, the current research assessed whether watching an educational animation that explains how EGMs work and subsequently setting a monetary limit prior to play via a pop-up message increases adherence to limits more than being exposed to either responsible gambling tool alone.

## Promoting Responsible Gambling

Increasingly, governments and the gambling industry are accepting responsibility for the implementation of public health oriented harm-reduction measures designed to minimize excessive gambling and its negative consequences across all strata of the general population (see Wohl et al. in press). These harm-reduction strategies aim to decrease the risks associated with gambling and facilitate responsible gambling, without overtly disturbing those who gamble in a non-problematic manner (Productivity Commission 2010). The ultimate decision to gamble and do so responsibly remains with the gambler. However, the gambler must be fully informed (i.e., educated) about how the games they play work, the probability of winning, and how to gamble responsibly. Subsequently, efforts on the part of government and the industry are required to encourage and assist gamblers, for example, to limit their monetary expenditure to within their discretionary disposable levels (Wohl et al. in press).

Although all forms of gambling can be associated with harm, EGMs have been identified as particularly problematic (Dowling et al. 2005). EGMs are disproportionately represented as the primary form of gambling reported by problem gamblers seeking treatment (Productivity Commission 2010). Among other reasons, this is due to the structural characteristics of EGMs. In particular, wins are positively reinforcing via classical and operant conditioning, which facilitates the development of problem gambling. In addition, the intermittent random ratio of reinforcement schedule that is inherent to EGMs is resistant to extinction, thus maintaining problematic play. Indeed, as a consequence of the reinforcement schedule, problem gamblers often experience cravings in anticipation of gambling as well as cravings to continue play once engaged (Young and Wohl 2009). The net effect is that EGMs encourage play and hinder gamblers ability to make informed decisions about limit setting as well as limit adherence (see Diskin and Hodgins 1999, 2001; O’Connor and Dickerson 2003; Stewart and Wohl in press).

With the aim of promoting informed decision making about responsible play, Wohl et al. (2010) created an animation that educated gamblers about the prudence of setting expenditure limits when playing as well as strategies to avoid exceeding those limits. Central to this animation was also the dispelling of EGM myths (e.g., the Jackpot becomes a more likely outcome the longer a person plays a particular EGM). Dispelling EGM myths is important from a responsible gambling perspective because erroneous cognitions and the misunderstanding of concepts linked to randomness and probabilities represent key components contributing to the initiation and maintenance of problem gambling in general, and EGMs in particular (Blaszczynski and Nower 2002; McCusker and Gettings 1997; Walker 1992; Wohl et al. 2005). Wohl et al. (2010) found that participants who watched the educational animation compared to a neutral video demonstrated a reduction in erroneous cognitions and perceived habits for limiting problematic play as effective. Importantly, following their next gambling session, participants reported staying within preset monetary limits more if they watched the educational animation than the neutral video. These results are encouraging but suggest that greater attention be devoted to setting expenditure limits during the animation.

Other researchers have examined the efficacy of signs and posters on, or in close proximity to, EGMs to educate gamblers on the importance of setting monetary limits when they gamble (e.g., Hing 2004; Manitoba Gaming Control Commission 2009). While awareness of such signage is relatively high, many gamblers report failing to read the content of the signs and posters, especially problem gamblers who deny they have a gambling problem (Hing 2004; Hing and Mattinson 2005). Moreover, simple signs providing gamblers with accurate information to correct erroneous cognitions and warn about the risks of gambling have been showed to be generally ineffective in promoting responsible gambling (Monaghan and Blaszczynski 2010b).

To be effective in increasing message comprehension and subsequent behavioral impact, signs need to attract attention. To this end, Monaghan and Blaszczynski (see Monaghan and Blaszczynski 2007; Monaghan 2008; Monaghan and Blaszczynski 2010a) have assessed the utility of intermittent messages (i.e., ‘pop-up messages’) that appear on EGM screens during play. They showed that such pop-up messages are noticed and recalled to a greater extent than static signs and displays. Furthermore, research with regular gamblers in gambling venues found that pop-up messages have a greater impact on thoughts and behavior resulting in greater awareness of session length, more breaks in play and shorter sessions compared to standard signs (Monaghan and Blaszczynski 2010a). These findings, however, are limited because no money was wagered as gamblers played on simulated gaming machines.

Research also indicates that gamblers typically do not believe that the information provided on responsible gambling signage applies to them, think that they have personal strategies to beat the odds (such as luck; Wohl and Enzle 2002, 2003), or do not believe or trust the information (Wohl et al. in press). As such, Stewart and Wohl (in press), sought to extend the work of Monaghan and Blaszczynski by assessing the utility of pop-up messages that explicitly ask gamblers to set a monetary limit prior to play and then remind gamblers when their limit has been reached. They found that failure to adhere to their preset monetary limit was positively associated with symptoms of gambling pathology. However, participants who received a pop-up reminder were significantly more likely to adhere to monetary limits than participants who did not. Dissociation mediated the relationship between gambling symptomatology and adherence to monetary limits, but only among those who did not receive a pop-up reminder. To the point, the pop-up reminder broke the dissociation that hindered limit adherence. Thus, akin to the educational animation, a pop-up message that first ask gamblers to set a monetary limit and then reminds them when that limit has been reached is a particularly effective responsible gambling tool.

## Assessing the Responsible Gambling Tools and their Relative Utility

Several countries, including Canada, New Zealand, Norway and Australia, have jurisdictions that introduced or are in the initial stages of introducing player card systems that enable gamblers to preset limits on their monetary expenditure in an effort to reduce impulsivity and encourage gambling within affordable means. Unfortunately, there is a lack of empirical evidence to guide the design of these systems. Although there is initial indication that educational animations and pop-up messages are independently effective means of facilitating limit setting and adherence, responsible gambling tools are rarely introduced into player card systems in isolation. Indeed, players are exposed to multiple responsible gambling features during play. For example, the MyPlay program used by the Nova Scotia Gaming Cooperation (NSGC) uses an array of responsible gambling tools. This system has been installed on all of NSGC’s video lottery terminals (VLTs) and aims at offering players tools, such as limits on money and time, as well as self-exclusion options and play history in order to promote responsible gambling.

Because efforts to implement harm minimization strategies to reduce the risks associated with gambling are on the rise, empirical assessment of possible additive or interactive effects of responsible gambling tools is required. Specifically, what is currently unclear is whether some responsible gambling tools are more effective than others or if use of more than one responsible gambling tool at a time has an additive effect on responsible gambling.

To this end, the current study aimed to empirically test the utility of two responsible gambling tools simultaneously—an educational animation and pop-up messages. It was hypothesized that participants who watched the educational animation would self-report significantly less erroneous cognition than those who watched the neutral video. This is because the animation directly targets erroneous cognitions. Given that the pop-up message does not target erroneous cognitions, we predicted that there would be no main effect of this variable or interaction with the animation. Lastly, akin to Wohl et al. (2010) initial assessment, participants should rate the animation as entertaining, of good quality, and educational.

It was also predicted that participants who were exposed to the monetary limit pop-up reminder would be better able to detect when they reached their limit than those who were not exposed to this reminder. Simply, this is because pop-up messages create a break in play and reminds the gambler when their preset monetary limit has been reached.

Given that research has yet to examine the potential interactive effect of exposure to an educational animation and pop-up messages on responsible play (i.e., limit adherence), no specific predictions were made. Instead, we point to some possible outcomes. First, watching the educational animation about how EGMs work and then being exposed to a monetary limit setting pop-up message might yield greater adherence to limits than if exposed to any one of these tools on their own. Specifically, gamblers might be more apt to set and adhere to a limit if they are exposed to multiple responsible gambling tools. It is also possible that one of the two tools is simply more effective at producing limit adherence than another. For example, reminding gamblers when their limit has been reached may improve limit adherence over and above any benefits drawn from watching an educational animation. That said, given the substantive role of erroneous cognitions in the development of gambling problems (Toneatto et al. 1997; Wohl et al. 2005), educational materials designed to identify and correct common erroneous cognitions may be especially useful in promoting responsible gambling. These possibilities were directly assessed in the current study.

## Method

### Participants

Participants consisted of 72 young adults (70.8 % female) enrolled in undergraduate courses at a large Canadian university. Participants’ ages ranged from 18 to 28 years (*M* = 19.69, *SD* = 1.82). As the primary purpose of the study was to assess the preventive potential of the animation and pop-up reminders, only recreational gamblers were recruited for the current study. Participants’ symptoms of gambling problems were known prior to the study because the 10-item gambling checklist from the Diagnostic and Statistical Manual of Mental Disorders IV (American Psychiatric Association 2000) was distributed to the student population at the onset of the academic year. This provided a measure of symptoms of gambling pathology over the past 12 months. All the people contacted agreed to participate in the present study. Upon entering the laboratory, the lack of symptoms of pathology was confirmed using the Problem Gambling Severity Index (PGSI) from the Canadian Problem Gambling Index (CPGI; Ferris and Wynne 2001). Specifically, using the PGSI, all participants were categorized as non-problem or low-risk gamblers (*M* = .68; *SD* = 1.03).

Participants represented mixed racial and ethnic backgrounds, including Caucasian (84.7 %), East Asian (4.2 %), Hispanic and South American (2.8 %), South Asian (1.4 %), African Canadian (1.4 %), and Native Canadian (1.4 %). An additional 4.2 % indicated other or multi-ethnic.

### Procedure

Participants arrived at the laboratory and were informed that the study involved assessing gambling behavior and that they would gamble on EGMs in a virtual reality (VR) casino. Written, informed consent was received before participants moved to a computer terminal and put on a head-mounted display (Z800 3D Visor, eMagin Corporation, Fishkill, New York) that enabled viewing of the VR environment. Participants acclimatized to the VR environment by navigating around a city environment, finding and entering the casino, and walking around the casino for five minutes.

Participants were given a total of 80 credits (at 25 cents each for a total of $20) to gamble with on the EGMs in the VR casino and informed that they had the chance to win more credits. Participants were instructed that they could trade any credits remaining at the end of the session for money, which they would be allowed to keep.

Prior to play, all participants watched a short 9-min video; in the animation condition, participants watched a custom-designed educational animation video. This video was designed to examine common misconceptions about how EGMs function and then correct these misconceptions in a cognitively simple manner. For example, the animation demonstrated that all outcomes were random and not linked to previous events (for a detailed description of this animation see Wohl et al. 2010). Participants in the neutral condition watched a 9-min video produced by Ontario Lottery and Gaming Corporation detailing their annual revenue from lotteries, lottery games offered, and the location of various casinos in Ontario. No mention was made about how EGMs function.

After watching one of the two videos, participants entered the VR casino and approached any vacant EGM. After money was inserted, a pop-up message appeared on the screen requiring participants to set a limit on the number of credits (out of their 80) they wished to spend on the EGM. After setting a limit participants were allowed to play as long as they wished.

The outcome of all EGM spins were pre-programmed so all participants experienced the same sequence of wins and losses. In the pop-up reminder condition, participants were informed via a pop-up message when their limit had been reached and asked them whether they wished to continue gambling (‘yes’ or ‘no). Regardless of their response, a second pop-up message instructed participants to inform the experimenter of this decision before proceeding. Participants were then given a battery of questionnaires to complete, which assessed demographics, gambling history, gambling severity and erroneous cognitions. Upon completing the questionnaires, participants who indicated that they wished to continue gambling were permitted to do so (the EGM was programmed such that participants would eventually lose all their 80 credits). In the no pop-up reminder condition, participants played until they wished to stop or they ran out of credits. Thereafter, they completed the same questionnaire battery.

All participants were fully debriefed at the end of the experimental session. They were compensated $30 for their time, regardless of how much money they had remaining at the end of the gambling session.

### Measures

#### Demographics and Gambling History

This questionnaire gathered background information from the participants (e.g., age, gender, and ethnicity). Additionally, all participants were asked about their gambling history including recent gambling experiences, average expenditures, and their game of choice.

#### Erroneous Cognitions

The 25-item Informational Biases Scale (IBS; Jefferson and Nicki 2003) is an established measure that assesses erroneous cognitions in relation to the nature of chance events, randomness, and independence of game outcomes (e.g., “I would rather use a EGM that I am familiar with than one that I have never used before”). Items are rated on a 7-point Likert scale, with 1 representing *‘Don’t agree at all’* and 7 representing *‘Strongly agree’.* Higher scores represent greater misperceptions of randomness, odds, and sampling with replacement. This scale demonstrated high internal reliability (α = .92) in the current study.

#### Limit Detection

Participants were asked the extent to which they were able to detect that they had reached their limit. This item was, “I noticed when I hit my limit.” This item was anchored at 1 (*strongly disagree*) and 7 (*strongly agree*).

#### Adherence to Preset Limit

Whether or not participants adhered to their preset monetary limit was tracked (0 = *stayed within preset limit*; 1 = *exceeded preset limit*).

## Results

### Preliminary Analyses

All participants reported prior gambling experience with EGMs. The vast majority (87.1 %) of participants reported setting monetary limits prior to gambling.

To assess whether age influenced how people responded to the manipulations, we regressed the animation condition variable (0 = neutral video; 1 = animation video), the pop-up condition variable (0 = no pop-up reminder; 1 = pop-up reminder), age, all two-way interaction terms, as well as the three-way interaction term on erroneous cognitions and limit detection. There were no significant main effects of age, *p*s > .25, and no significant interactions (two- or three-way) with age, *p*s > .09. Logistic regression with the same predictor variables on adherence to preset limits indicated that there were no significant main effect of age, *p* > .28, and no significant interactions (two- or three-way) with age, *p*s > .10. Therefore, we collapsed across age for all subsequent analyses.

We then conducted a series of three-way between-groups Analysis of Variances (ANOVAs; Animation Condition x Pop-up Condition x Symptomatology) that showed neither main effects of problem gambling symptomatology (using the PGSI), *p*s > .26, nor significant interactions (two- or three-way), *p*s > .11, on erroneous cognitions or limit detection. Logistic regression with the same predictor variables on adherence to preset limits indicated neither a main effect of problem gambling symptomatology, *p*s > .09, nor significant interactions (two- or three-way), *p*s > .13. Therefore, we collapsed across problem gambling symptomatology for all subsequent analyses.

Gender differences regarding the pathway toward gambling pathology may vary as a function of gender-based differences in the manner to which they appraise gambling situations (see Matheson et al. 2009; Volberg 2002). A series of three-way between-groups ANOVAs (Animation Condition x Pop-up Condition x Gender) were conducted on erroneous cognitions and limit detection. Neither the main effects of gender, *p*s > .08, nor the interactions involving gender, *p*s > .10, were significant for either dependent variable. Logistic regression with the same predictor variables on adherence to preset limits indicated neither a main effect of gender, *p*s > .95, nor significant interactions (two- or three-way), *p*s > .08. Therefore, we collapsed across gender for all subsequent analyses.

Lastly, a 2 (animation manipulation) × 2 (pop-up manipulation) ANOVA was conducted on the monetary limit participants’ set prior to play. There were neither significant main effects of the animation manipulation, *F*(1,69) = .37, *p* = .55, η_p_² = .005 or the pop-up manipulation, *F*(1,69) = 3.10, *p* = .08, η_p_² = .04, nor a significant interaction, *F*(1,69) = 1.85, *p* = .18, η_p_² = .03. Thus, participants set statistically equivalent limits across conditions.

### Measured Variables

Means and standard deviations for all measured variables by condition are reported in Table 1 as well as the number of participants per condition.

**Table 1:** Means and Standard Deviations for measured variables by condition

	Educational animation		Neutral video	
	Pop-up no Reminder (n = 20)	Pop-up Reminder (n = 17)	Pop-up Reminder (n = 17)	No Pop-up Reminder (n = 18)
Erroneous cognitions				
Mean (SD)	2.17 (.87)_a_	2.31 (1.01)_a_	3.17 (1.10)_b_	3.04 (1.16)_b_
Limit detection				
Mean (SD)	6.21 (1.51)_a_	4.75 (1.84)_b_	5.35 (1.66)_ab_	4.67 (2.25)_b_

Comparisons in a given row with different subscripts are significantly different at *p* < .05. Numbers in parentheses are standard deviations

#### Erroneous Cognitions

A 2 (animation manipulation) × 2 (pop-up manipulation) ANOVA was applied to the erroneous cognitions variable. There was a main effect of the animation manipulation, *F*(1,68) = 12.52, *p* = .001, η_p_² = .16. As predicted, participants who watched the animation had significantly fewer erroneous cognitions (*M* = 2.23, *SD* = .92) than participants who watched the neutral video (*M* = 3.10, *SD* = 1.12). As expected, there was neither a main effect of the pop-up manipulation, *F*(1,68) = .01, *p* = .98, η_p_² < .001, nor a significant interaction, *F*(1,68) = .744, *p* = .35, *η*_*p*_ *²* = .005 on erroneous cognitions.

#### Limit Detection

A 2 (animation manipulation) × 2 (pop-up manipulation) ANOVA was applied to the limit detection variable. Although there was not a significant main effect of the animation manipulation, *F*(1,66) = 1.15, *p* = .29, η_p_² = .02, there was a significant main effect of the pop-up manipulation, *F*(1,66) = 5.97, *p* = .02, η_p_² = .08. As predicted, participants who were exposed to a pop-up reminder were better able to detect when they reached their limit (*M* = 5.81, *SD* = 1.62) than participants who were not exposed to a reminder (*M* = 4.71, *SD* = 2.04). No significant interaction was observed, *F*(1,66) = .78, *p* = .38, *η*_*p*_ *²* = .01.

### Adherence to Monetary Limits Set Prior to Electronic Gaming Machine Play

Chi-square analysis was used to determine differences in categorical data. A three-way frequency analysis was conducted to examine the degree of association between (1) the animation manipulation, (2) the pop-up manipulation, and (3) adherence to pre-set limits. Backward stepwise elimination was used to develop hierarchical log-linear models and determine the model of best fit. Log-linear analysis is a generalization of the χ^2^ analysis which allows the researcher to examine the impact of more than one categorical variable together as well as the interactions of each variable in modeling the data (see Tabachnick and Fidell 2001).

Analysis revealed that both the animation and monetary limit pop-up reminder helped facilitate adherence to preset limits, χ^2^ (1) = 6.83, *p* = .009 and χ^2^ (1) = 6.83, *p* = .009, respectively. Specifically, participants who watched the animation adhered to their preset limit more (97 %) than those who watched the neutral video (77 %). Likewise, participants who were provided a reminder of their preset monetary limit adhered to that limit more (97 %) than those who were not reminded (77 %).

**Fig. 1:**
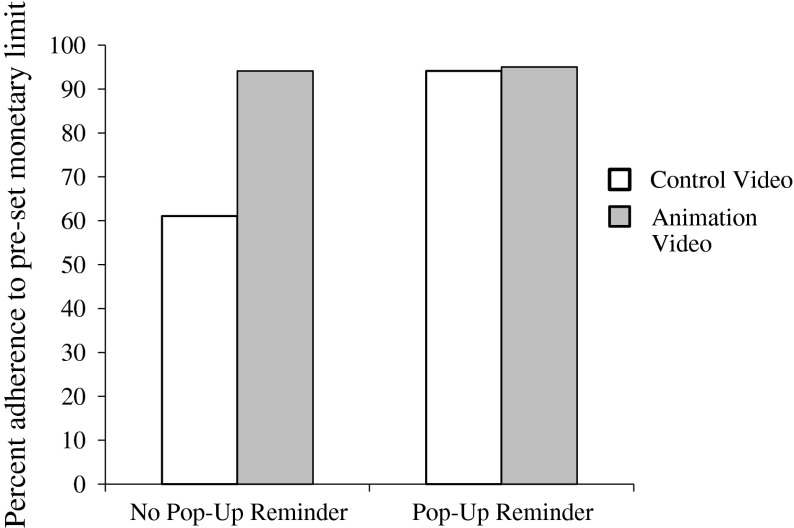
Adherence to pre-set monetary limit (in percentage) by animation and pop-up reminder conditions

However, as depicted in Fig. 1, the two main effects were qualified by an interaction between the two manipulated variables on limit adherence.^2^ Among participants who did not experience the pop-up reminder, those who watched the animation stayed within their pre-set limits (94.1 %) more than those who did not watch the animation (61.1 %), χ^2^(1) = 5.40, *p* = .02. Odds ratios indicated that among those who did not receive the pop-up, those who watched the neutral video were 10 times more likely to exceed their limit compared to those who watched the animation video. Among participants who were reminded of their limit via pop-up message, however, there was no difference in limit adherence between those who watched the animation (95 %) and those who did not watch the animation (94.1 %), χ^2^(1) = .01, *p* = .91.

### Impressions of the Animation

Wohl et al.’s (2010) initial assessment of the animation used a community sample of gamblers who reported a positive impression of the animation. In the current study, a university student sample was employed. Thus, we thought it prudent to examine if, akin to the community sample, young adults had a positive impression of the animation. To this end, we asked participants in the animation condition whether they found the animation entertaining (*M* = 5.10, *SD* = 1.22), of good quality (*M* = 5.15, *SD* = 1.10), and educational (*M* = 5.23, SD = 1.61) on a scale anchored at 1 (*strongly disagree*) and 7 (*strongly agree*). The means for all responses were significantly above the midpoint of the scale, *p*s < .001. Because participants who viewed the animation ranged in age from 18 to 28, we also assessed whether age predicted their impression of the animation. Regression analyses showed that age did not predict impression on any of the measured variables, *p* > .27

## Discussion

Increasingly, gambling jurisdictions are examining avenues to assist their patrons to gamble responsibly. As a result, there has been a rapid increase in the production of various tools to facilitate responsible gambling. Unfortunately, few responsible gambling tools have been empirically tested resulting in a significant gap in the research available to guide public policy. Two tools that have received some empirical attention are educational animations that explain how EGMs function and pop-up messages that help guide the gambler to play responsibly (Monaghan 2008; Monaghan and Blaszczynski 2007; Monaghan and Blaszczynski 2010a; Stewart and Wohl in press; Wohl et al. 2010). However, the extant empirical research has focused on assessing the utility of one type of responsible gambling tool at a time. In all responsible gambling systems that have been implemented, however, gamblers are exposed to an array of tools. In the current study, we assessed the effect of exposure to both an educational animation and a pop-up message in the same gambling session.

Consistent with predictions, participants who viewed the educational animation reported significantly less gambling-related erroneous cognition than those who viewed the neutral video. This finding confirms Wohl et al. (2010) initial examination of the efficacy of this responsible gambling tool, which found that the educational animation was associated with a decrease in erroneous cognitions. Importantly, the current research also extends previous findings by assessing adherence to preset monetary limits in vivo—the previous investigation with the animation asked participants to retrospectively report on limit adherence. We showed that the pro-responsible gambling effect of the educational animation on limit adherence obtained by Wohl et al. (2010) was not simply due to motivated recall among those who viewed the animation. Indeed, viewing the animation resulted in greater adherence to preset monetary limits during the monitored laboratory gambling session.

We also showed that participants who were exposed to a monetary limit pop-up reminder during EGM play were more aware of when they had reached their limit than participants who were not exposed to a reminder. This result provides support for the effectiveness of monetary limit pop-up messages coupled with reminders as a strategy that can be used to encourage responsible gambling (see Stewart and Wohl in press). When a gambler intensely focuses on and becomes absorbed in play they tend to lose track of time and space, thus undermining the monetary limit set prior to initiating play (see Diskin and Hodgins 1999, 2001; Jacobs 1988; Wynne 1994). The consequence is that gamblers are often not aware that they have exceeded their preset monetary limit (Stewart and Wohl in press). The pop-up message reminds the gambler that their limit has been reached thus enhancing the prospect of limit adherence and, by extension, responsible gambling.

Of central concern for the current research, however, was the possible additive or interactive effect of exposure to more than one responsible gambling tool within a given session. Indeed, in the current study, an interaction was observed that qualified the two main effects. Among participants who did not experience the pop-up reminder, those who watched the animation stayed within their monetary preset limit more than those who did not watch the animation. For those who were reminded of their monetary limit, however, there was no difference in limit adherence between those who watched the animation and those who watched the neutral video. Thus, there was no additive effect of exposure to both responsible gambling tools. Both the educational animation and monetary limit pop-up messages were found to aid in the adherence to a preset monetary limit.

It is possible that a ceiling effect is reached with a pop-up reminder of the gambler’s preset limit. Indeed, the vast majority of participants in both the educational animation and neutral video condition who received a monetary limit pop-up reminder adhered to their monetary limit. These results suggest it would behove policy makers to focus their responsible gambling attention (though not undivided) on the relatively unobtrusive pop-up message. It is an effective means of increasing preset limit adherence, which could have significant downstream effects on gambling-related harm reduction.

## Limitations

Some limitations of the current research should be noted. First, the sample consisted of university-recruited young adult gamblers and as such, results of the current research may not generalize to other populations. Gainsbury and colleagues (see Gainsbury and Blaszczynski 2011; Gainsbury et al. in press) have discussed the limitations of conducting research with this population and concluded that as a result of participant characteristics, results may inflate the potential impact of responsible gambling tools. However, as increased rates of at-risk and problem gambling have been documented among university students and young adults (Barnes et al. 2010; Welte et al. 2011) it was considered useful to examine the efficacy of the responsible gambling tools in this population. However, it is important that this research be replicated with a sample of gamblers more representative of the general population.

It should also be noted that the current study was conducted in a laboratory setting rather than an actual gambling venue. Further, participants were not wagering their own money. Although attempts were made to increase the salience of the potential winnings, it is possible that they were not playing as they would with their own money. Consequently, results may be limited in the extent to which they can be generalized to a session of EGM gambling in a real gambling venue. However, it has been found that if appropriate factors are introduced, such as the chance to win or lose money, the gambling experience in a VR environment is similar to that in a real casino (Leary and Dickerson 1985). With respect to the current study, results regarding the educational animation closely mimic those of Wohl et al. (2010) who assessed gamblers in a casino and who were betting with their own funds. Further, by conducting the current research in a laboratory setting, we were able to control for extraneous variables (e.g., win and losses) and randomly assign participants to experimental conditions. This allowed for greater causal inferences to be drawn and increased internal validity. Finally, future research should examine the impact of mandatory limit setting on EGM play. It is possible that simply being required to set a limit prior to play may increase adherence to that limit.

## Implications

Despite the methodological limitations noted, the current research has important implications for the prevention of problematic EGM play. Specifically, by highlighting the fact that EGM wins are random, unpredictable and beyond personal control, the educational animation appears to be effective in decreasing erroneous cognitions held by non-problem EGM gamblers. Moreover, it appears that by stressing the importance of setting and adhering to a preset monetary limit, the educational animation encourages gamblers to adhere to a monetary limit set prior to play. Given that the educational animation was associated with a decrease in erroneous cognitions and exceeding a monetary limit, this responsible gambling tool may be particularly effective in reducing the risk of problem gambling among EGM players. Importantly from an uptake perspective, people in both the current study and Wohl et al. (2010) found the educational animation to be enjoyable, of good quality, and (perhaps most importunately) educational. Similar impressions should thus be expected if the gambling industry were to include this video in their suite of responsible gambling tools.

A monetary limit pop-up reminder also appeared to be highly effective at promoting responsible play. By briefly suspending play to remind gamblers when they reach their preset limit, pop-up reminders informed players of when their preset limit was reached. In doing so, pop-up messages encourage EGM players to gamble within their limit and reduce the risk of problematic EGM play.

The effect of the brief pop-up reminder appeared to be equally effective as the educational animation. This suggests that this brief intervention could be highly effective and minimally intrusive for gamblers. Given that the aim of responsible gambling strategies is to minimize disruption among recreational gamblers, the pop-up message required less time commitment than viewing the educational animation and thus might be a preferred strategy if time is an issue. With that said, it is possible that pop-up messages may lose their effectiveness over time. Specifically, gamblers may become desensitized to the pop-up with repeated exposer (see Daryanani 2011). Future research should test this possibility with a longitudinal study.

## Conclusion

The results of the current research have immediate applied significance for policy makers. Specifically, a pop-up message reminding players they have reached their preset limit should be implemented for land-based and online EGMs to encourage responsible gambling and reduce the potential risks associated with EGM gambling. However, this responsible gambling tool is only effective to the extent that gamblers have the ability to set a monetary limit prior to play. Several jurisdictions (e.g., Australia and Canada) are moving towards requiring limit setting to be a mandatory feature for land-based EGMs and online gambling sites. Further evaluation of limit setting in gambling venues should be conducted to validate various responsible gambling tools—both in isolation as well as part of a holistic strategy.

## Footnotes

1 It is recognized that gambling within means is a subjective sum of money and likely dependent on the personal wealth of the gambler. Although some people can afford to lose substantial sums of money they may still develop problem gambling symptomatology (e.g., lying about gambling and neglecting important activities and relationships)—they are simply able to avoid or delay some negative problem gambling-related outcomes due to their financial circumstances.

2 Although no three-way interaction was detected with the log-linear analysis, possible two-way interactions were probed to assess the viability of this possibility.
